# Effect of Glutaraldehyde-Based Desensitizer on Control of Tooth Sensitivity and Tooth Color Post-bleaching: A Randomized Clinical Trial

**DOI:** 10.1055/s-0044-1789603

**Published:** 2024-12-10

**Authors:** Raíssa Araújo de Mesquita, Elma Vieira Takeuchi, Maria Eduarda Cardoso de Oliveira Pereira, Jesuina Lamartine Nogueira Araújo, Eliane Bermeguy Alves, Cecy Martins Silva

**Affiliations:** 1 Department of Restorative Dentistry, School of Dentistry, Federal University of Pará, Belém, PA, Brazil

**Keywords:** tooth bleaching, dentin desensitizing agents, dentin sensitivity, glutaraldehyde

## Abstract

**Objectives** This randomized, controlled, single-blind, split-mouth clinical study evaluated the effect of the application of a glutaraldehyde-based desensitizer on the prevention of tooth sensitivity (TS) and the changes in the color of the teeth after bleaching.

**Materials and Methods** Twenty-five patients were selected for participation in the study according to the inclusion and exclusion criteria. The patients' right and left hemiarches were randomized into two groups: the placebo group, which received distilled water application and whitening treatment, and the Gluma group, which received Gluma application, followed by whitening treatment. The patients were examined after three bleaching sessions with 35% hydrogen peroxide. TS was measured using a numerical rating scale for 21 days from the beginning of bleaching. The tooth color was monitored using a spectrophotometer at times T0 (baseline) and T1 (after 21 days). The color analysis results were recorded using the CieLab system; the CIEDE2000 formula was applied to obtain the ΔE ^00^ and ΔL values.

**Statistical Analysis** For statistical analysis, the Friedman analysis of variance test was used for intragroup evaluation, and the Wilcoxon test was used for a between-group comparison of the TS results. Student's *t* -test paired the ΔE ^00^ and ΔL values of the groups. A 5% significance level was adopted.

**Results** Intragroup analysis of the sensitivity results showed a statistically significant difference between the pain levels evaluated through days 1 to 21 ( *p* < 0.001), and the highest median values were observed on the days when the whitening sessions were performed (days 1, 8, and 15) and right after the sessions (days 2, 9, and 16). However, in the intergroup analysis, no statistical difference in sensitivity was found between the placebo and Gluma. No statistical difference was found between the influences of the placebo and Gluma treatments on the color obtained after tooth whitening using parameters ᐃE ^00^ and ᐃL ( *p* > 0.05).

**Conclusion** The use of Gluma prior to bleaching does not prevent TS and does not interfere with the color results obtained by tooth bleaching.

## Introduction

Improvements in aesthetic procedures in dental clinics are constantly being sought. Studies report that 20 to 30% of the evaluated populations are not satisfied with the color of their teeth and want to make them whiter. 1 2 Dental bleaching is a conservative, simple, low-investment, and successful therapy for this purpose 3 and is one of the most common procedures in aesthetic dentistry. 4 However, a systematic review has shown that tooth bleaching often results in tooth sensitivity (TS). 5 Thus, clinicians and researchers are developing strategies for its prevention. 6 7

Post-bleaching TS has been reported as the result of reversible inflammation of the dental pulp due to hydrogen peroxide and the free radicals resulting from the reaction, which permeate the enamel and dentin, and can penetrate the chemo sensitive ion channels (TRPA1) present in the pulp. 8 9

Several randomized clinical trials have been carried out to analyze post-bleaching TS prevention options. Systemic use of analgesic and anti-inflammatory drugs has not yielded satisfactory results. 10 11 12 However, topical application of desensitizers is considered effective for this type of transient sensitivity. 13 14 The use of various products, such as potassium nitrate, 15 strontium chloride, 16 remineralizing agents, 17 and photobiomodulation, 18 and the association between therapies 19 20 have shown very positive results for the control of TS induced by tooth whitening.

Products based on glutaraldehyde and hydroxyethyl methacrylate-HEMA, such as Gluma Desensitizer (Heraeus, Kulzer-Hanau, Germany), are widely used. Their mechanism of action involves agglomerations that occur through the reaction of glutaraldehyde to the proteins present in the dentinal tubules, leading to a reduction in their diameters. 21 22 These plugs are formed when HEMA facilitates glutaraldehyde's penetration of the dentin, which can lead to tubular obliteration.

Few studies have evaluated the post-bleaching TS reduction effect of Gluma. 21 22 Moreover, the results obtained have been controversial. While one study obtained a significant reduction in TS when the product was used prior to bleaching, 21 the other did not demonstrate a satisfactory action. 22 However, an experimental gel based on glutaraldehyde and potassium nitrate obtained satisfactory results in the prevention of post-bleaching TS. 23

Considering the scarce literature on Gluma Desensitizer and the controversial results obtained by the few studies that have been carried out on it, this randomized clinical study was conducted to evaluate the effect of the glutaraldehyde-based desensitizer on TS and the color obtained after tooth whitening. The following were the proposed null hypotheses:

H01: There will be no difference in TS reduction between the group treated with a glutaraldehyde-based desensitizing agent and the placebo group after tooth whitening.
H02: There will be no difference in tooth color change between the group treated with a glutaraldehyde-based desensitizing agent and the placebo group after tooth whitening.

## Materials and Methods

### Ethical Aspects

The present clinical study followed the guidelines of the Consolidated Standards of Reporting Trials (CONSORT) 24 was registered on the public website of clinical trial records (Clinicaltrials.gov; number: NCT05309967) and was approved by the Ethics Committee for Research in Human Beings of the Institute of Health and Sciences of the Federal University of Para (CEP-ICS/UFPA no. 5.680.18). The participants were instructed about the study objectives, design, and risks in accordance with the Declaration of Helsinki. 25

### Study Design

The present study was a randomized, blinded, split-mouth, placebo-controlled clinical trial. Within the period from March 2021 to December 2022 at the Faculty of Dentistry of the Federal University of Pará in Belém, Brazil, the participants were randomly assigned to either the placebo or Gluma groups and received either of the two protocols for each hemiarch.

### Sample Size

The sample size of the study was defined based on a previous pilot study that used the same method proposed in this investigation. The GPower 3.1 program (Heinrich-Heine-Universität Düsseldorf, Germany) was used. Eighty percent statistical power, 5% α error, and 20% end-of-study sample loss prediction were adopted. The calculated sample size for this study was 20 patients, with an additional 5 patients to account for potential loss, totaling 25 patients per group. The validity of these values was confirmed by previous studies. 18 19

### Inclusion and Exclusion Criteria

The participants selected for the trial were 18 to 35 years of age; either male or female; with healthy incisors, canines, and premolars; without dentin hypersensitivity (DH); and had not undergone treatment with desensitizers in the 12 months before the start of the study. The diagnosis of hypersensitivity was made through anamnesis and clinical examination, and only patients who did not report dentin sensitivity to the evaporative stimulus or had no previous history of DH were included in the study.

Patients undergoing orthodontic treatment, who had periodontal diseases or severe incidents of dental darkening, or who were pregnant and/or breastfeeding women were excluded from the study.

### Randomization and Allocation Concealment

The randomization process to allocate each side of mouth to either Gluma or placebo groups was performed using the Bioestat 5.0 software (Sociedade Civil, Mamirauá, Pará, Brazil), which used a computer-generated random table. The data were placed in sealed envelopes that were opened by the operator on the first day of the conduct of the protocol.

### Blinding

The study was considered a single-blind one; that is, the evaluator, who analyzed the results, was not aware of the randomization process and allocation of the studied groups. The participants and operator were also not aware of the randomization process, and the desensitizers were placed in identical containers to prevent identification. However, as the tested product has a characteristic odor, the blinding could not have been total.

### Study Intervention

Prior to the tooth whitening procedure, the selected patients received standardized oral hygiene kits with dentifrices that did not have whitening or desensitizing agents (My First Colgate-Colgate Palmolive Company), in addition to brushing instructions.

The sessions started with prophylaxis with a rubber cup pumice and construction of the gingival barrier in both arches (Top Dam, FGM, Joinville, SC, Brazil).

#### Desensitizing Treatment

Desensitizing treatment was performed prior to dental whitening, one hemiarch at a time, respecting the randomization of the sides.

In the placebo group, distilled water was actively applied with a Microbrush (FGM-Joinville, SC, Brazil) for 10 seconds, after which 60 seconds were allowed to pass. The tooth surface was then dried with a jet of air until it no longer shined. This was followed by rinsing with water for 10 seconds.

Gluma Desensitizer was applied to the Gluma group according to the manufacturer's instructions, using the same procedure as with the placebo group.

#### Whitening

Whitening with 35% hydrogen peroxide (Whitness Hp, FGM, Joinville, SC, Brazil) was performed with three 15-minute applications, totaling 45 minutes for each session. Three whitening sessions were held, with 7-day intervals.

#### Sensitivity Evaluation

To assess the participants' perceptions of pain, they were asked to complete a questionnaire daily for 21 days after the first whitening treatment session. A numerical rating scale (NRS) was used as a reference, in which 0 corresponds to total absence of pain; 1 to 3, mild pain; 4 to 7, moderate pain; and 8 to 10, severe pain.

#### Color Change Analysis

The colors of the participants' teeth were recorded by a single operator, with the aid of a Vita Easyshade Advance Spectrophotometer (Vita Zahnfabrink, Germany), at the following times: T0 (baseline) and T1 (21 days after the first session). The area of interest for the color registration was the middle third of the central incisors and canine teeth's buccal surfaces, which were standardized using a customized matrix made with laboratory condensation silicon (Zetalabor-Zhermack SpA, Badia, Italy).

The color analysis results were recorded by the CieLab system using the CIEDE2000 method to obtain the ΔE ^00^ e ΔL data. To obtain ∆E ^00^ data comparing the beginning and end of treatment, data from L, C, H, a*, and b* at times T0 and T1 were used for two teeth in each group (maxillary central incisor and canine), and the values were included in the CIEDE2000 formula, as follows 25 26 :

∆E ^00^ = [(∆l'/KLSL)2 + (∆C'/KCSC)2 + (∆H'/KHSH)2 + RT(∆C'/KCSC)(∆H'/KHSH)2]1/2,

where ΔL, ΔC, and ΔH are the differences in lightness, chroma, and hue, respectively, at each pair of points. The weighting functions (SL, SC, and SH) adjust the total color difference by the variations in the L', a', and b' coordinate pairs. The parametric factors k _l_ , k _c_ , and k _h_ are correction terms for experimental situations. For calculation purposes, all parametric factors were set to 1 ((k _l_ = k _c_ = k _h_ = 1).

The rotation function (RT) accounts for the interaction between the chroma and hue differences in the blue region. 26 27

### Statistical Analysis

The obtained data were computed in Microsoft Excel tables and analyzed using Jamovi statistical software Version 1.6 (Computer Software, Sydney, Australia). After assessing the normality of the data distribution, the intragroup sensitivity results were evaluated using the Friedman analysis of variance test, and for intergroup comparisons, the Wilcoxon test was used at each time point. Color assessment was performed using the paired Student's *t* -test to compare ΔE ^00^ and ΔL between the two groups. A 5% significance level and 80% power were adopted.

## Results

### Participants

**Fig. 1:**
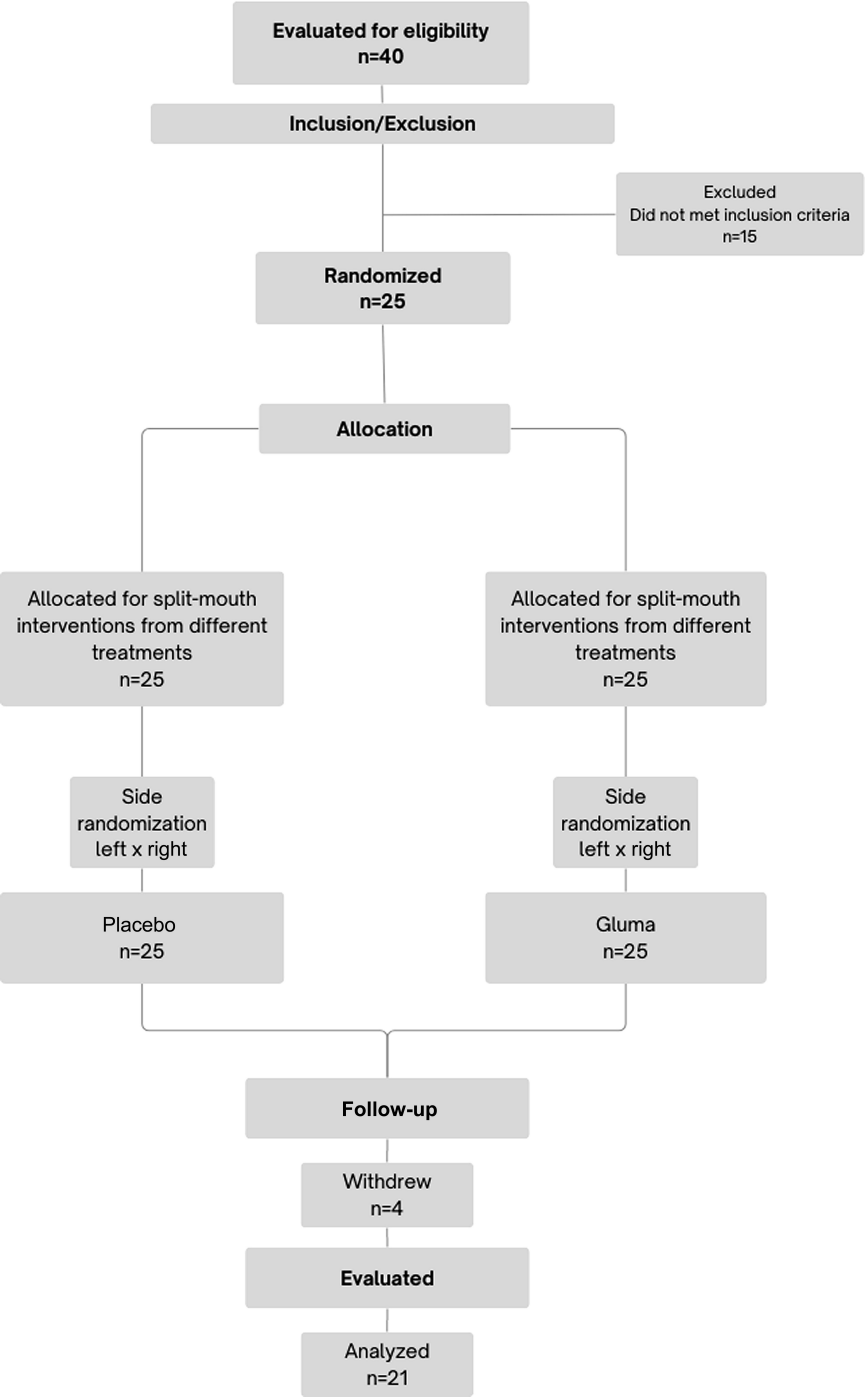
Flow diagram of the study design phases according to the inclusion and allocation criteria.

Forty volunteers were evaluated for sample selection, 15 of whom were excluded for not meeting the inclusion and exclusion criteria. The remaining 25 were randomized, treated, and followed up according to Fig. 1 . Two participants withdrew after the first session and two withdrew after the second session. Thus, 21 patients completed the study, and the data obtained from them were analyzed.

### Demographic Characteristics

As the study was a split-mouth study, the study groups' male–female distributions did not show a significant difference ( *p* = 0.275), and the mean age between the groups was the same ( Table 1 ).

**Table 1:** Demographic characteristics

	Gluma	Placebo	*p* -Value
**Gender** Male, ***n*** (%) Female, ***n*** (%)	8	8	** 0.275 a**
	13	13	
**Age** Mean (standard deviation)	25.7 (± 4.12)	25.7 (± 4.12)	**NaN**

a McNemar test.

### Sensitivity Analysis

In the intragroup analysis of the sensitivity results, a statistically significant difference was shown between the pain levels evaluated through days 1 to 21 ( *p* < 0.001), and the highest median values were observed on the days when the whitening sessions were performed (days 1, 8, and 15) and on days 2, 9, and 16, the days after the sessions. However, in the intergroup analysis, no statistical difference in sensitivity was found between the placebo and Gluma groups ( *p* > 0.05; Table 2 ).

**Table 2:** Median and interquartile deviation of sensitivity recorded in the T21 questionnaire

Md (±IQR)							
Week 1	Day 1	Day 2	Day 3	Day 4	Day 5	Day 6	Day 7
** Placebo**	3(4)Aa	0.5(1.5)Ba	0(0)Ca	0(0)Ca	0(0)Ca	0(0)Ca	0(0)Ca
** Gluma**	3(3.5)Aa	0.5(1.5)Ba	0(0.5)Ca	0(0)Ca	0(0)Ca	0(0)Ca	0(0)Ca
**Week 2**	Day 8	Day 9	Day 10	Day 11	Day 12	Day 13	Day 14
** Placebo**	3.5(3)Aa	1(2.5)Ba	0(0)Ca	0(0)Ca	0(0)Ca	0(0)Ca	0(0)Ca
** Gluma**	4(3)Aa	1(2.5)Ba	0(0)Ca	0(0)Ca	0(0)Ca	0(0)Ca	0(0)Ca
**Week 3**	Day 15	Day 16	Day 17	Day 18	Day 19	Day 20	Day 21
** Placebo**	4(6)Aa	0.5(3)Ba	0(0)Ca	0(0)Ca	0(0) Ca	0(0)Ca	0(0)Ca
** Gluma**	4(4)Aa	0.5(3)Ba	0(0)Ca	0(0)Ca	0(0)Ca	0(0)Ca	0(0)Ca

Abbreviations: IQR, interquartile range; Md, median

Notes: The capital letters represent the intragroup statistical difference for the Friedman analysis of variance test ( *p* ≤ 0.05). The lowercase letters represent the statistically significant differences in the intergroup evaluation (Wilcoxon test; *p* ≤ 0.05).

### Color Change Analysis

No statistical difference was found between the influences of the placebo and Gluma treatments on the color obtained by whitening determined using parameters ᐃE ^00^ and ᐃL ( *p* > 0.05; Table 3 ).

**Table 3:** Mean and standard deviation of the ᐃE ^00^ and ᐃL values after tooth whitening

M (± SD)			
	Gluma	Placebo	*p* -Value
** ᐃE ^00^**	4.22 (± 1.27)	4.13 (± 1.37)	0.827
**ᐃL**	2.83 (± 2.96)	3.27 (± 2.14)	0.585

Abbreviations: M, mean; SD, standard deviation.

Notes: Reference value: *p* ≤ 0.05. Paired Student's *t* -test was used to perform comparisons between the evaluated groups.

## Discussion

Post-bleaching sensitivity is the primary adverse effect reported by patients who have undergone tooth whitening treatment and may limit the number of patients opting for 13 or completing it. 20 Otherwise, tooth whitening is widely requested by patients, and there is evidence that it can enhance the quality of life of those individuals. 28 Protocols for preventing or reducing TS after this procedure have been much discussed in the literature. The main objective of the present study was to evaluate whether Gluma Desensitizer could prevent sensitivity caused by whitening and, as a secondary objective, whether it would interfere with the color result obtained.

Gluma Desensitizer is an adhesive liquid desensitizer composed of glutaraldehyde and 2-hydroxyethyl methacrylate-HEMA. It has the capacity to penetrate the dentinal tubules and reduce the painful sensation. 11 29 30 Its mechanism of action suggests that the application of glutaraldehyde on the dental surface causes the precipitation of plasma proteins, such as albumins, and these occlude the tubules, interfering with the hydrodynamics of the dentinal fluids. 21 22 These coagulated proteins allow the polymerization of HEMA and the formation of plugs that close the dentinal tubules. HEMA also facilitates glutaraldehyde penetration to a depth of 200 μm, considerably decreasing dentin permeability. 31

Dental enamel is generally considered permeable as the free radicals generated by hydrogen peroxide (molar mass: 34 g/mol) can penetrate enamel and dentin after 5- to 15-minute application. 22 Thus, some previous studies have suggested that the active ingredients of Gluma (glutaraldehyde [molar mass: 100 g/mol] and HEMA [molar mass: 130 g/mol]) could also diffuse through enamel and dentin through the same path used by the free radicals of hydrogen peroxide. 21 22 However, no previous studies have shown the effectiveness of this diffusion, indicating that more studies should be conducted to determine how Gluma Desensitizer penetrates the enamel to reach the dentin.

The randomized clinical study conducted by Mehta et al 21 reported a positive outcome for Gluma compared with placebo, which contrasts with the findings of the present study. The former study utilized a gel presentation (Gluma Power Gel, Kulzer, Germany) with a composition similar to that of Gluma Desensitizer liquid. However, the whitening protocol in Mehta et al's study involved a single session with a 15-minute application of 40% hydrogen peroxide. In contrast, the present study employed a protocol consisting of three sessions, each involving a 45-minute application of 35% hydrogen peroxide. Therefore, it can be inferred that the hydrogen peroxide application, in accordance with the manufacturer's guidance, used in the present study was considerably more intensive than in Mehta et al's study, potentially leading to a higher level of sensitivity that the desensitizer could not mitigate. It has been reported that the longer the hydrogen peroxide application time, the greater the damage to the pulp complex and the observed sensitivity. 22 32 Kose et al 32 concluded that the sensitivity in a group in which hydrogen peroxide application was performed only once for 15 minutes was lower than that in the groups with three-time 15-minute hydrogen peroxide application.

Mohamed and Banna 33 evaluated the use of glutaraldehyde and other desensitizing agents prior to bleaching in patients with DH and found a reduction in sensitivity after Gluma application. However, their study was not placebo-controlled and used a different method than the present clinical trial, which only assessed post-bleaching sensitivity. In the former study, a sensitivity analysis was performed before and immediately after the application of desensitizers and the bleaching procedure. Consequently, there was no isolated assessment of sensitivity following the application of the desensitizing agents studied. Therefore, the positive results obtained by Mohamed and Banna 33 may suggest that desensitizing agents, such as Gluma, are effective in controlling DH. However, their findings do not clearly demonstrate that Gluma influences the sensitivity caused by tooth whitening.

Diniz et al 22 performed a similar method to that in the present study and no reduction in sensitivity was observed when Gluma was used prior to tooth whitening. This may be due to the incomplete obliteration of dentinal tubules promoted by the product, as it does not appear to be total. 34 35 Instead, only a reduction in the diameter of these tubules may occur, which will still allow the passage of hydrogen peroxide to the pulp tissue. In addition, hydrogen peroxide can help dissolve the precipitated material, consequently reducing the effectiveness of the desensitizer when used prior to bleaching. It is known that Gluma Desensitizer can immediately reduce sensitivity, 11 but in previous studies, 36 37 a significant decrease in sensitivity was observed 10 minutes after its application, an action that was also disclosed by the manufacturer. 38 It can be inferred that a decrease in sensitivity after Gluma application can be expected only after a few minutes, when protein plugs have been formed inside the dentinal tubules.

Based on the results obtained by this study that was compared with other scientific findings, the null hypothesis 01 cannot be rejected.

Self-reported sensitivity assessment is the most commonly used method for measuring pain. The NRS is well accepted and generates data that can be audited, 39 which is why this method was chosen for the present study. A split-mouth study design was used as it allows for greater homogeneity among participants, with patients servings as their own controls. Consequently, the number of participants needed to demonstrate statistical efficiency is reduced. 40 To maintain the maximum homogeneity of the sample, we provided to all patients hygiene kits and delivered oral hygiene instructions prior to the clinical interventions. Dietary instructions or restrictions were not provided, as recent evidence suggests that color restrictions are not necessary. 41

Both study groups showed effectiveness in tooth whitening. Therefore, hypothesis 02 was accepted. In the CIEDE2000 method, the ᐃE ^00^ values represent the color change between the hue, chroma, and value parameters, 27 and ᐃL determines the difference in luminosity. 26 The present study's results showed that both groups obtained representative color change values. This confirmed the results obtained in previous studies, 21 22 in which the use of glutaraldehyde with HEMA prior to whitening enabled a satisfactory and equivalent color result, unlike placebo. Therefore, Gluma did not interfere with hydrogen peroxide's penetration of the pulp tissue as it allowed effective whitening. If dentin permeability and hydrogen peroxide penetration are considered the main causes of postbleaching sensitivity, then the number of applications of the desensitizing agent Gluma may have been a limitation of the present study. Three applications might not have been sufficient to prevent the adverse effects of hydrogen peroxide penetration into the pulp tissue. Additionally, the inability to conduct a double-blind study, partly due to the distinct odor of the desensitizer tested and the difficulty in creating a placebo with similar characteristics, is another limitation of this study. Therefore, it is suggested that further clinical studies should be conducted with a larger sample size, double-blinding, and a greater number of product applications before or after whitening to better understand efficacy of Gluma in combination with hydrogen peroxide.

## Conclusion

Despite the limitations of the current study, it can be concluded that Gluma Desensitizer did not effectively prevent postwhitening sensitivity. In addition, it demonstrated no significance difference in color results when comparing placebo and Gluma groups.

## References

[1] Shulman J D Maupome G Clark D C Levy S M Perceptions of desirable tooth color among parents, dentists and children J Am Dent Assoc 2004 135 05 595 604, quiz 654–655 15202751 10.14219/jada.archive.2004.0247

[2] Alkhatib M N Holt R Bedi R Prevalence of self-assessed tooth discolouration in the United Kingdom J Dent 2004 32 07 561 566 15386863 10.1016/j.jdent.2004.06.002

[3] Rezende M Loguercio A D Kossatz S Reis A Predictive factors on the efficacy and risk/intensity of tooth sensitivity of dental bleaching: a multi regression and logistic analysis J Dent 2016 45 1 6 26612623 10.1016/j.jdent.2015.11.003

[4] Kwon S R Wertz P W Review of the mechanism of tooth whitening J Esthet Restor Dent 2015 27 05 240 257 25969131 10.1111/jerd.12152

[5] Cardenas A FM Maran B M Araújo L CR Are combined bleaching techniques better than their sole application? A systematic review and meta-analysis Clin Oral Investig 2019 23 10 3673 3689 10.1007/s00784-019-03042-4 31468261

[6] Kwon S R Dawson D V Schenck D M Fiegel J Wertz P W Spectrophotometric evaluation of potassium nitrate penetration into the pulp cavity Oper Dent 2015 40 06 614 621 26151563 10.2341/14-214-L

[7] Klaric Sever E Budimir Z Cerovac M Clinical and patient reported outcomes of bleaching effectiveness Acta Odontol Scand 2018 76 01 30 38 28893130 10.1080/00016357.2017.1376111

[8] Caviedes-Bucheli J Ariza-García G Restrepo-Méndez S Ríos-Osorio N Lombana N Muñoz H R The effect of tooth bleaching on substance P expression in human dental pulp J Endod 2008 34 12 1462 1465 19026874 10.1016/j.joen.2008.09.013

[9] Costa C A Riehl H Kina J F Sacono N T Hebling J Human pulp responses to in-office tooth bleaching Oral Surg Oral Med Oral Pathol Oral Radiol Endod 2010 109 04 e59 e64 10.1016/j.tripleo.2009.12.002 20303048

[10] Almassri H NS Zhang Q Yang X Wu X The effect of oral anti-inflammatory drugs on reducing tooth sensitivity due to in-office dental bleaching: a systematic review and meta-analysis J Am Dent Assoc 2019 150 10 e145 e157 31561766 10.1016/j.adaj.2019.05.023

[11] Coppla F M Rezende M de Paula E Combination of acetaminophen/codeine analgesics does not avoid bleaching-induced tooth sensitivity: a randomized, triple-blind two-center clinical trial Oper Dent 2018 43 02 E53 E63 29504880 10.2341/17-092-C

[12] Rezende M Bonafé E Vochikovski L Pre- and postoperative dexamethasone does not reduce bleaching-induced tooth sensitivity: a randomized, triple-masked clinical trial J Am Dent Assoc 2016 147 01 41 49 26562735 10.1016/j.adaj.2015.07.003

[13] Wang Y Gao J Jiang T Liang S Zhou Y Matis B A Evaluation of the efficacy of potassium nitrate and sodium fluoride as desensitizing agents during tooth bleaching treatment—a systematic review and meta-analysis J Dent 2015 43 08 913 923 25913140 10.1016/j.jdent.2015.03.015

[14] Krishnakumar K Tandale A Mehta V Post-operative sensitivity and color change due to in-office bleaching with the prior use of different desensitizing agents: a systematic review Cureus 2022 14 04 e24028 35547454 10.7759/cureus.24028 PMC9090214

[15] Martins L M Lima E Souza L A Sutil E Clinical effects of desensitizing prefilled disposable trays in in-office bleaching: a randomized single-blind clinical trial Oper Dent 2020 45 01 E1 E10 31891544 10.2341/18-149-C

[16] de Melo Alencar C Castro da Silva R Nogueira Araújo J L Silva da Silveira A D Martins Silva C A clinical, randomized, double-blind study on the use of toothpastes immediately after at-home tooth bleaching Am J Dent 2017 30 05 267 271 29178730

[17] Alexandrino L D Alencar C M Silveira A DSD Alves E B Silva C M Randomized clinical trial of the effect of NovaMin and CPP-ACPF in combination with dental bleaching J Appl Oral Sci 2017 25 03 335 340 28678953 10.1590/1678-7757-2016-0408 PMC5482257

[18] Moosavi H Arjmand N Ahrari F Zakeri M Maleknejad F Effect of low-level laser therapy on tooth sensitivity induced by in-office bleaching Lasers Med Sci 2016 31 04 713 719 26964798 10.1007/s10103-016-1913-z

[19] de Paula B Alencar C Ortiz M Couto R Araújo J Silva C Effect of photobiomodulation with low-level laser therapy combined with potassium nitrate on controlling post-bleaching tooth sensitivity: clinical, randomized, controlled, double-blind, and split-mouth study Clin Oral Investig 2019 23 06 2723 2732 10.1007/s00784-018-2715-4 30361793

[20] Pompeu D DS de Paula B LF Barros A PO Combination of strontium chloride and photobiomodulation in the control of tooth sensitivity post-bleaching: a split-mouth randomized clinical trial PLoS One 2021 16 04 e0250501 33909659 10.1371/journal.pone.0250501 PMC8081218

[21] Mehta D Venkata S Naganath M LingaReddy U Ishihata H Finger W J Clinical trial of tooth desensitization prior to in-office bleaching Eur J Oral Sci 2013 121 05 477 481 24028597 10.1111/eos.12067

[22] Diniz A Lima S Tavarez R Preventive use of a resin-based desensitizer containing glutaraldehyde on tooth sensitivity caused by in-office bleaching: a randomized, single-blind clinical trial Oper Dent 2018 43 05 472 481 29570018 10.2341/17-020-C

[23] Parreiras S O Szesz A L Coppla F M Effect of an experimental desensitizing agent on reduction of bleaching-induced tooth sensitivity: a triple-blind randomized clinical trial J Am Dent Assoc 2018 149 04 281 290 29439773 10.1016/j.adaj.2017.10.025

[24] CONSORT Group Schulz K F Altman D G Moher D CONSORT 2010 statement: updated guidelines for reporting parallel group randomized trials Ann Intern Med 2010 152 11 726 732 20335313 10.7326/0003-4819-152-11-201006010-00232

[25] World Medical Association World Medical Association Declaration of Helsinki: ethical principles for medical research involving human subjects JAMA 2013 310 20 2191 2194 24141714 10.1001/jama.2013.281053

[26] Durand L B Ruiz-López J Perez B G Color, lightness, chroma, hue, and translucency adjustment potential of resin composites using CIEDE2000 color difference formula J Esthet Restor Dent 2021 33 06 836 843 33283966 10.1111/jerd.12689

[27] Sharma G Wu W Dalal E The CIEDE2000 color-difference formula: implementation notes, supplementary test data, and mathematical observations Color Res Appl 2005 30 01 21 30

[28] Fiorillo L Laino L De Stefano R Dental whitening gels: strengths and weaknesses of an increasingly used method Gels 2019 5 03 35 31277412 10.3390/gels5030035 PMC6787621

[29] Lopes A O de Paula Eduardo C Aranha A CC Evaluation of different treatment protocols for dentin hypersensitivity: an 18-month randomized clinical trial Lasers Med Sci 2017 32 05 1023 1030 28391435 10.1007/s10103-017-2203-0

[30] Lopes A O Aranha A C Comparative evaluation of the effects of Nd:YAG laser and a desensitizer agent on the treatment of dentin hypersensitivity: a clinical study Photomed Laser Surg 2013 31 03 132 138 23421629 10.1089/pho.2012.3386 PMC3589893

[31] Qin C Xu J Zhang Y Spectroscopic investigation of the function of aqueous 2-hydroxyethylmethacrylate/glutaraldehyde solution as a dentin desensitizer Eur J Oral Sci 2006 114 04 354 359 16911108 10.1111/j.1600-0722.2006.00382.x

[32] Kose C Calixto A L Bauer J R Reis A Loguercio A D Comparison of the effects of in-office bleaching times on whitening and tooth sensitivity: a single blind, randomized clinical trial Oper Dent 2016 41 02 138 145 26509229 10.2341/15-085-C

[33] Mohamed A I Banna M E Evaluation of different desensitizing agents after in-office bleaching Life Sci J 2011 8 01 164 168

[34] Öncü E Karabekiroğlu S Ünlü N Effects of different desensitizers and lasers on dentine tubules: an in-vitro analysis Microsc Res Tech 2017 80 07 737 744 28251725 10.1002/jemt.22859

[35] Fu B Shen Y Wang H Hannig M Sealing ability of dentin adhesives/desensitizer Oper Dent 2007 32 05 496 503 17910227 10.2341/06-143

[36] Abuzinadah S H Alhaddad A J A randomized clinical trial of dentin hypersensitivity reduction over one month after a single topical application of comparable materials Sci Rep 2021 11 01 6793 33762645 10.1038/s41598-021-86258-3 PMC7991651

[37] Mehta D Gowda V S Santosh A Finger W J Sasaki K Randomized controlled clinical trial on the efficacy of dentin desensitizing agents Acta Odontol Scand 2014 72 08 936 941 24909155 10.3109/00016357.2014.923112

[38] Heraeus Kulzer GmbH. Frequent asked questions [official website]. Accessed February 20, 2024 at:https://kulzer.com.br/pt/downloads/gluma_5/gluma_desensitizer_2/FAQs_-_GLUMA_Desensitizer.pdf

[39] Karcioglu O Topacoglu H Dikme O Dikme O A systematic review of the pain scales in adults: which to use? Am J Emerg Med 2018 36 04 707 714 29321111 10.1016/j.ajem.2018.01.008

[40] ISCB Subcommittee on Dentistry Lesaffre E Garcia Zattera M J Redmond C Huber H Needleman I Reported methodological quality of split-mouth studies J Clin Periodontol 2007 34 09 756 761 17716311 10.1111/j.1600-051X.2007.01118.x

[41] Hardan L Bourgi R Flores-Ledesma A Is a white diet necessary for tooth bleaching procedures? A systematic review and meta-analysis Dent J 2024 12 04 118 135 10.3390/dj12040118 PMC11049513 38668030

